# Some pleural effusions labeled as idiopathic could be produced by the inhalation of silica

**DOI:** 10.1515/pp-2021-0135

**Published:** 2022-01-03

**Authors:** Silvia Bielsa, Ana Guitart, Aureli Esquerda, Rodrigo Fernández-Pacheco, Maria Teresa Baranguán, Alfonso Ibarra, José M. Porcel

**Affiliations:** Department of Internal Medicine, Pleural Medicine Unit, Arnau de Vilanova University Hospital, Biomedical Research Institute of Lleida (IRBLleida), University of Lleida, Lleida, Spain; Chemical Analysis Service, Science Faculty, Zaragoza University, Zaragoza, Spain; Department of Laboratory Medicine, Arnau de Vilanova University Hospital, IRBLleida, Lleida, Spain; Aragon Nanoscience Institute, Advanced Microscopy Laboratory, Zaragoza University, Zaragoza, Spain

**Keywords:** idiopathic pleural effusion, occupational disease, pleural effusion, silica nanoparticles

## Abstract

**Objectives:**

Exposure to silica nanoparticles has been associated with pleural effusions (PEs) in animal models and case series. We hypothesized that some PEs labelled as “idiopathic” could, in fact, be secondary to inhalation of silica.

**Methods:**

A retrospective case control study was designed utilizing a prospectively maintained pleural database. Cases, represented by idiopathic PEs, were matched by age and gender to control patients who had been diagnosed with malignant, cardiac, or infectious PEs. A survey consisting of questions about occupational life and possibility of silica inhalation was conducted. In a subgroup of patients, pleural fluid concentrations of silica were quantified by plasma atomic emission spectrometry analysis. Also, the pleural biopsy of a silica-exposed case was subjected to an energy dispersive X-ray spectroscopy (EDX) to identify the mineral, the size of which was determined by electron microscopy.

**Results:**

A total of 118 patients (59 cases and 59 controls) completed the survey. There were 25 (42%, 95% CI 31–55%) and 13 (22%, 95% CI 13–34%) silica-exposed workers in case and control groups, respectively. The exposure attributable fraction was 0.62 (95% CI 0.14–0.83). Four of eight exposed cases showed detectable levels of silica in the pleural fluid (mean 2.37 mg/L), as compared to none of 16 tested controls. Silica nanoparticles of 6–7 nm were identified in the pleural biopsy of an exposed case patient.

**Conclusions:**

It is plausible that some idiopathic PEs could actually be caused by occupational silica inhalation.

## Introduction

Occupational exposure to inhaled particles, such as silica and silicate, has been associated with the development of lung inflammation and occupational disease [1]. Song et al. reported a group of seven patients who were exposed to polyacrylate spray paint and developed pleural effusion (PE), inflammation, fibrosis, and foreign body granulomas in the pleura [2]. In a subsequent analysis of the pleural fluid (PF) samples, silica nanoparticles of 20 nm were detected [3]. Afterwards, these clinical findings were replicated in a rat model of polyacrylate/nanosilica nanoparticles which were instilled intratracheally, thus supporting the hypothesis that inhaled silica nanoparticles can generate PEs [4, 5]. Even though both animal and *in-vitro* pleural models with other nanoparticles, such as carbon nanotubes [6, 7] or silver [8, 9], have demonstrated several pathological changes in pleural tissues, no studies thus far have investigated whether exposure to silica may be the underlying cause of some PEs originally labelled as “idiopathic”. Occupational settings where inhalation of silica may occur include brickwork or any exposure to sand, crushed stone, paints, ceramics, abrasive powders, cements, or industrial/paint aerosols. Manufactured silica, sometimes in the form of nanoparticles, has broad applications in the paint and coating industry because it improves some characteristics of the industrial materials (e.g., strength, chemical resistance, and thermal stability). These artificial silica particles have been linked to adverse pleural effects [3].

We aimed to investigate whether some PEs considered to be of idiopathic causes could, in fact, be secondary to inhalation of silica. The starting point of this investigation was the discovery of silica nanoparticles in pleural tissue biopsies of a patient who developed a PE after the acute inhalation of a paint spray in an occupational setting.

## Materials and methods

### Setting and study design

A registry, which includes all consecutive patients with PE subjected to a diagnostic thoracentesis, has been prospectively maintained for the last two decades at our hospital. As of January 2016, the time point of this study design, the database contained 3,838 patients. A case control study was devised where cases that included “idiopathic” PEs were matched with randomly selected controls by age, gender, and year of diagnosis. Control patients had been diagnosed with PEs secondary to cancer, heart failure or other known etiologies. All proposed cases and selected controls for our study were contacted by phone to provide consent participation, unless they had died or were uncontactable. In the first part of the study, they were interviewed regarding their occupational exposure to silica. The second part consisted of analyzing the concentrations of silica in the PF, which included a randomly selected subgroup of cases and controls with PF samples stored in our biobank. Finally, we obtained more precise data on the silica nanoparticles in the pleural tissue of the patient who originally motivated the current research. The study was approved by the local Ethics Committee (CEIC, University Hospital Arnau de Vilanova, ID 1592-2016).

### Diagnostic criteria for pleural effusions

The diagnosis of malignant PEs was established by demonstrating malignant cells in either PF or pleural biopsy specimens. Identification of PE due to heart failure was based on the clinical history, physical examination, chest radiographs, electrocardiogram, echocardiogram (if available) and positive response to diuretics. Parapneumonic PEs were exudative effusions associated with pneumonia, bronchiectasis, or pulmonary abscesses. Pleural tuberculosis was confirmed through the isolation of *Mycobacterium tuberculosis* from sputum, PF or pleural biopsy samples or, alternatively, the demonstration of granulomas in pleural tissue. Finally, a PE was classified as idiopathic when it remained undiagnosed despite a study that had included PF analyses (biochemistry, culture, and cytology), chest and abdominal computed tomography and, in patients with PEs of sufficient size, pleural biopsy. Idiopathic PEs were always followed up for a minimum of two years to rule out relapses or new diagnoses. Other PE etiologies were established according to widely accepted clinical criteria.

### Telephone survey on occupational exposure

From January to April 2016, a telephone survey was conducted for all cases and selected controls. Patients were invited to respond to a questionnaire about their working life (Supplementary Material, Annex 1), after which they were classified into five categories: 1) homemakers/janitors/kitchen workers, 2) agricultural or animal husbandry, 3) skilled laborer, such as bricklayer/carpenter/mechanic/miner/painter, 4) office worker or similar, and 5) others.

The questionnaire covered demographic characteristics (sex and age); occupation; exposure to sand, crushed stone, paints, ceramics, abrasive powders, cements, or aerosols; exposure time; and use of protective measures like breathing masks. We considered patients to be “occupationally exposed” if they belonged to the third group or had been exposed to any of the aforementioned materials. The interviewer was blinded of the PE etiology.

### Measurement of silica concentrations in pleural fluid

Our center stores PF samples in a biobank from most patients subjected to a PF tap, particularly since 2000. PF samples are collected into tubes containing EDTA, centrifuged and stored at −80 °C in 1 mL aliquots until analyzed. Silica concentrations in PF were quantified by plasma atomic emission spectrometry analysis (Supplementary Material, Annex 2). The minimum detectable level of silica is 0.059 mg/L.

### Analysis of silica nanoparticles on pleural tissue

In one patient who underwent a diagnostic thoracoscopy, the biopsy samples were analyzed using transmission electron microscopy according to standard protocols. Specifically, biopsy fragments were immediately embedded into 5% glutaraldehyde overnight. Tissue was cut into ultrathin sections and stained with uranyl acetate and lead citrate. The samples were examined in a Tecnai F30 Field Emission Gun transmission electron microscopy (TEM), operating at 300 kV, with scanning transmission electron microscopy (STEM) mode. A nanometric probe was formed and the sample was scanned point by point. High Angle Annular Dark Field (HAADF) images were obtained pixel by pixel. To identify the chemical elements that were present in the sample, an energy dispersive X-ray (EDX) analysis was performed on different regions of the biopsy tissue using an EDX detector.

### Statistical analysis

Categorical variables were expressed as absolute numbers (percentages) and continuous variables as medians (25 and 75th percentiles). To determine the overall impact of the potential association of silica exposure and PE we calculated exposure-attributable (EAF) and population-attributable (PAF) fractions. EAF is the proportion of incidents (i.e., PEs) in the exposed group that are attributable to the risk factor (i.e., exposure to silica), whereas PAF refers to the proportion of disease incidence in a population that is attributable to an exposure. After calculating the PAF and the prevalence of exposure among the controls, sample size and statistical power were estimated according to the study of Browner and Newman [10]. Assuming a PAF of 0.26 and a prevalence of 0.2 in the control group, the minimum number of patients to be reached in each group was between 50 and 60. We used p<0.05 (2-tailed) to determine statistical significance. Statistical calculations were performed using the SPSS statistical package version 24.0 (Chicago, IL, USA).

## Results

### Case-control study

The case population included patients who could be contacted by phone, specifically 59 of 103 (57%) who had a final diagnosis of idiopathic PE, according to our database. The control population also comprised 59 patients, of whom 20 had malignant PEs from different primaries (lung 12, unknown origin 2, ovary 2, and 1 each of breast, gastric, endometrial, and lymphoma), 19 heart failure, 9 parapneumonics, 7 post-abdominal surgery, 2 tuberculosis, 1 post-trauma, and 1 Sjögren’s syndrome. In each group, the median age of patients was 68 (53–78) years, and 37 (62%) were men.

Table 1 shows the distribution of patients in each occupational group. Twenty-five cases (42%, 95% CI 31–55%) and 13 controls (22%, 95% CI 13–34%; p=0.018) were deemed to be exposed to silica. The EAF and PAF were 0.62 (95% CI 0.14–0.83) and 0.26 (95% CI 0.12–0.49), respectively.

**Table 1:** Occupational groups among cases and controls.

Variable	Cases, n=59	Controls, n=59	p-Value
Current or previous professions			
Homemakers, janitors, or kitchen workers	9 (15%)^a^	10 (17%)	
Agricultural or animal husbandry	11 (19%)	14 (24%)	
Skilled laborers^b^	22 (37%)	13 (20%)	
Office worker or similar	16 (27%)	17 (29%)	
Other	1 (2%)^c^	5 (8%)^d^	
Occupational exposure to silica	25 (42%, 95% CI 31–55)	13 (22%, 95% CI 13–34)	0.018
Odds ratio of occupational exposure	2.6 (95% CI 1.2–5.8)		–
EAF	0.62 (95% CI 0.14–0.83)		–
PAF	0.26 (95% CI 0.12–0.49)		–

^a^Two were industrial cleaners with aerosol contact. ^b^Bricklayer/carpenter/mechanic/miner/painter. ^c^Sand factory. ^d^Two truck drivers, and one each electrical technician, watchmaker, and long-term unemployed. EAF, exposure-attributable fraction; PAF, population-attributable fraction.

Table 2 shows the data for the subgroup of 52 working-age patients (less than 65 years). Twelve (46%, 95% CI 29–65%) and five (19%, 95% CI 9–38%; p=0.038) subjects from the case and control groups, respectively, had a history of silica exposure. The EAF and PAF were 0.72 (95% CI 0.4–0.92) and 0.33 (95% CI 0.13–0.63), respectively.

**Table 2:** Distribution of occupations among subjects with active working age (<65 years).

Variable name	Cases, n=26	Controls, n=26	p-Value
Actual or previous professions			
Homemakers, janitors, and kitchen workers	5 (19%)^a^	3 (12%)	
Agricultural or animal husbandry	2 (8%)	4 (5%)	
Skilled laborers^b^	10 (38%)	5 (19%)	
Office worker or similar	9 (35%)	9 (35%)	
Other	0	5 (27%)^c^	
Occupational exposure to silica	12 (46%, 95% CI 29–65)	5 (19%, 95% CI 9–38)	0.038
Odds ratio of occupational exposure	3.6 (95% CI 1.04–12.48)		
EAF	0.72 (95% CI 0.04–0.92)		
PAF	0.33 (95% CI 0.13–0.63)		

^a^Two were industrial cleaners with aerosol contact. ^b^Bricklayer/carpenter/mechanic/miner/painter. ^c^Two truck drivers and one each electrical technician, watchmaker, and long-term unemployed. EAF, exposure-attributable fraction; PAF, population-attributable fraction.

Twelve (32%, 95% CI 19–47%) of the 38 silica-exposed patients reported the use of protective masks at work, but only one actually wore a filter mask. The wearing time of the mask was also questioned. One patient wore the mask 50% of the time at work, while the remaining 11 patients used it between 5 and 30% of the time. Only one patient who used a protective mask belonged to the control group. The median exposure time to silica was 20 years (quartiles 6–34 years) in the case group and 27 years (quartiles 10–34 years) in the control group.

### Silica concentrations in pleural fluid

**Figure 1:**
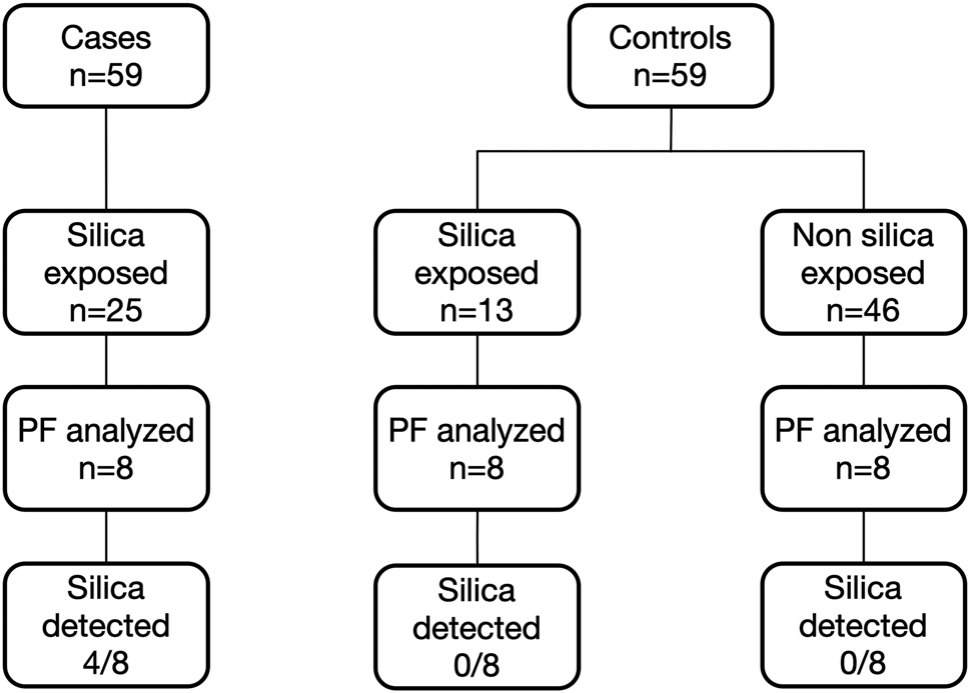
Diagram of selected patients for the analysis of the presence of pleural fluid silica.

Figure 1 represents a diagram of patients whose PFs were analyzed to detect silica. Only eight of the 25 exposed cases (idiopathic PEs) had stored PF for analysis. We randomly selected 16 control patients (i.e., with a known cause of PE), of whom eight had a history of occupational exposure to silica and eight did not. PF spectrometry analysis showed that four of the eight exposed cases had detectable levels of silica in PF (mean 2.37 mg/L, standard deviation 2.52 mg/L) (Table 3), as compared with none of the 16 control patients (p=0.01).

**Table 3:** Characteristics of patients with detectable silica levels in pleural fluid.

Sex and age, years	Job	Exposure time, years	Type of protective mask and time using it at work	Pleural biopsy method	PF silica, mg/L, mean ± SD
Man, 40	Bricklayer	4	Cotton, 50%	Thoracoscopy	4.902 ± 0.423
Man, 37	Paint factory	4	Cotton, 10%	Ultrasound-guided	4.174 ± 0.768
Man, 61	Airplane mechanic	12	Cotton, 5%	Thoracoscopy	0.313 ± 0.077
Man, 60	Pottery factory	5	No	Not performed	0.1 ± 0.022

PF, pleural fluid; SD, standard deviation of the silica amount.

Three of the four cases with detectable PF levels of silica wore protective masks (in particular, cotton face-breathing masks) and used them 50, 10 and 5% of the working time. None of the four exposed case patients without detectable levels of silica used protective masks.

Serum and PF parameters were compared between exposed cases, exposed controls, and non-exposed controls. Other than PF silica concentrations, the only significant difference between groups was a higher level of serum eosinophils in the former (Table 4). More than half of patients in each group had PEs that occupied one-third or less of the hemithorax; and two thirds of PEs were right-sided on chest radiographs.

**Table 4:** Serum and pleural fluid parameters in patients who underwent silica evaluation in pleural fluid and/or biopsy specimens.

	Exposed cases		Control patients		p-Value
	Silica positive	Silica negative	Exposed	Non-exposed	
Number of patients	5^a^	4	8	8	
Age, years	40 (35–61)	63 (44–66)	53 (42–64)	50 (39–65)	0.454
Silica levels, mean ± SD	2.37 ± 2.52	0 ± 0	0 ± 0	0 ± 0	0.01
Serum leukocytes/µL	9,980 (7,655–14,338)	9,005 (7,388–11,913)	10,905 (6,923–11,205)	9,445 (7,925–13,635)	0.966
Serum neutrophils/µL	6,185 (4,860–6,970)	5,575 (3,935–9,578)	7,550 (4,750–8,493)	6,915 (5,510–11,768)	0.585
Serum lymphocytes/µL	2,850 (1,485–4,973)	2,305 (1,338–2,605)	1,420 (1,008–2003)	1,455 (783–1700)	0.103
Serum eosinophils/µL	700 (300–1,160)^b^	280 (190–475)	190 (155–370)	40 (10–283)	0.025
PF erythrocytes/µL	2,300 (1,000–21,050)	11,300 (2,248–19,475)	6,650 (2,550–36,900)	15,550 (1,625–136,675)	0.77
PF leukocytes/µL	3,400 (1,272–7,303)	1,621 (594–11,800)	1,048 (207–3,291)	2,317 (677–5,837)	0.39
PF neutrophils/µL	208 (142–314)	100 (16–6,874)	469 (104–987)	701 (74–4,865)	0.603
PF lymphocytes/µL	3,128 (637–7,039)	1,387 (496–4,940)	443 (322–2,489)	882 (258–2,521)	0.415
PF eosinophils >10%	2 (40)	1 (25)	0 (0)	0 (0)	0.085

Data are presented as median (interquartile range) or number (percentages) unless otherwise specified. ^a^Including one patient with demonstrable silica on pleural biopsy, in whom no PF was available for silica detection. ^b^Significantly higher than the respective values in other groups by post-hoc test. PF, pleural fluid; SD, standard deviation.

### EDX study

**Figure 2:**
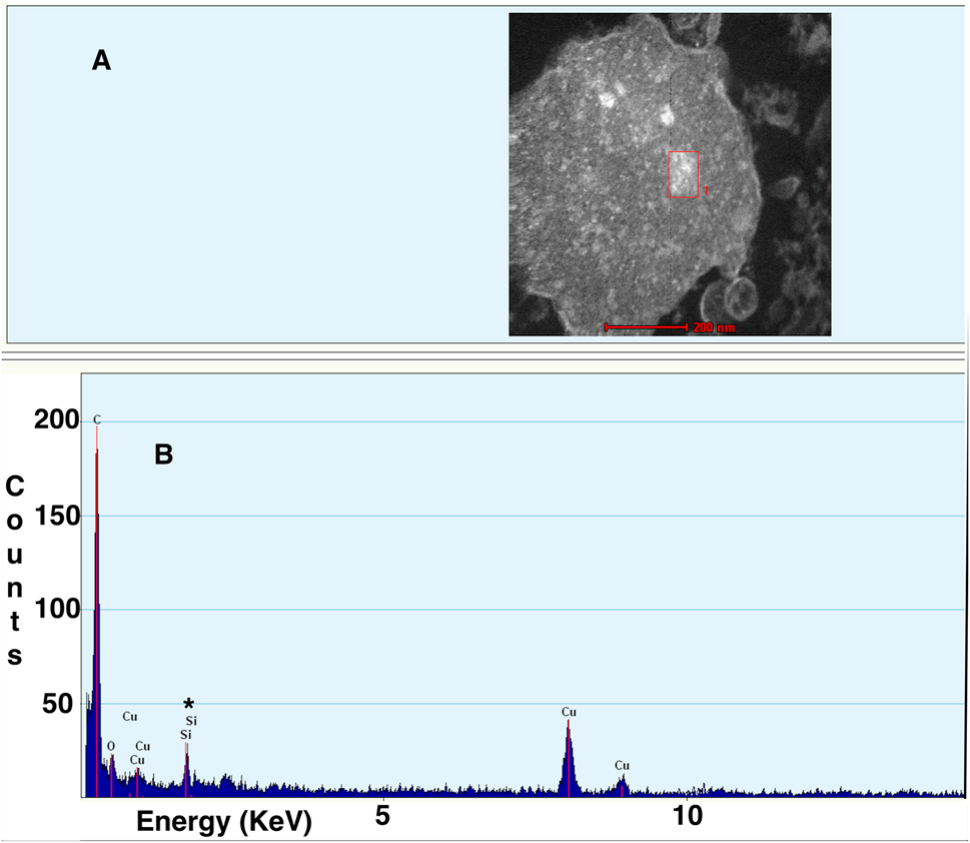
Energy dispersive X-ray analysis of a pleural tissue specimen.(A) Transmission electron microscopy images of silica nanoparticles obtained from pleural tissue (scale bar=200 nm). (B) Energy dispersive X-ray analysis revealed silica nanoparticles (Si) and peaks of carbon (C), oxygen (O), and copper (Cu). The copper readings in the Figure were from the grid holding the sample, not the sample itself.

In one patient with an idiopathic PE who was acutely exposed to paint spray, lung and pleural tissue samples were obtained by medical thoracoscopy. A pathological study of the pleura showed a fibrous thickening with fibrinous and inflammatory cells. Mild fibrosis in the lung tissue biopsy was also observed. When the pleural and lung biopsies were analyzed using electron microscopy, 6–7 nm nanoparticles were identified in the pleural tissue. The EDX analysis confirmed that these nanoparticles contained silica (Figure 2). However, no silica was detected by EDX analysis in the lung tissue.

## Discussion

This study suggests that occupational exposure to silica could be the underlying etiology of some idiopathic PEs. The value of the EAF (62%), the detection of PF levels of silica only in exposed case patients with idiopathic PEs, and the finding of silica nanoparticles in the pleural biopsy of an exposed case support this assertion. It can be argued that silica was found in just four of the 16 patients whose occupation had a high risk of particle inhalation. However, eight of them had well-established causes of the PE, thus implying the finding of silica in the PF of half the patients with occupational exposure who had previously been diagnosed of idiopathic PEs. If silica had been found in some exposed controls with a definite cause of PE, then its theoretical contribution to PE development would be questionable.

Song et al. reported a group of seven patients who were exposed to polyacrylate spray paint in a poorly ventilated workplace for 5–13 months [2]. All developed PEs and two died. The lung and pleural pathological examinations displayed nonspecific inflammation. Possibly, these patients would have been diagnosed as idiopathic PEs if they had not occurred concurrently in a specific work environment. Afterwards, using electron microscopy and EDX analysis, 20 nm silica nanoparticles were identified in lung and pleural biopsies [3]. Moreover, in a rat model the intratracheal instillation of polyacrilate/nanosilica was able to induce the appearance of new PEs, thus suggesting a causal relationship [5].

Silicosis, which is a common occupational lung disease secondary to exposure to silica dust after an appropriate latency, only presents with PE infrequently [11]. Even so, in an autopsy series of 110 silicosis, pleural thickening was found in 64 (58%) cases [12]. The hypothesis is that silicosis and PEs secondary to silica result from different pathophysiological mechanisms. Firstly, crystalline silicas are naturally occurring minerals that are ubiquitous in the earth’s crust [13] and the size of respirable dust ranges between 0.3 and 4.8 μm [14]. However, the reported 6–7 nm nanoparticles in our study are usually manufactured, especially in the paint and coating industry. Secondly, silicosis is a slowly progressive disease that usually takes more than 10 years to develop [15], while in our study and the Song et al. series [2] patients were younger and environmentally exposed for fewer than 10 years. However, there are case series reporting outbreaks of acute and accelerated silicosis related to artificial crystalline silica particles (quartz conglomerate) which are used to fabricate kitchen and bathroom countertops [16, 17].

In recent years, science has developed the ability to manipulate matter at the nanoscale level. However, nanomaterials present potential safety concerns and there is accumulating evidence to suggest that nanoparticles may exert adverse effects on the human organism [18]. As a result of their small size and different physicochemical properties, the toxic profiles of nanoparticles may differ considerably from those of larger particles which make up the same materials [18]. Kaewamatawong et al. compared the acute pulmonary toxicity induced in mice by the intratracheal administration of either colloidal amorphous silica nanoparticles (14 nm) or fine particles (213 nm) [19]. The nanoparticles had a greater ability to induce lung inflammation and tissue damage than the larger particles.

Ferrer et al. found silica by EDX analysis with scanning electron microscopy in the visceral pleura of necropsy specimens from 11 city dwellers with no clinical evidence of lung disease, 10 subjects occupationally exposed to silica without pneumoconiosis, and eight patients with silicosis [20]. Pleural silica content in silicosis patients was higher than in those without lung disease. The finding of the mineral in the visceral pleura was attributed to mechanical progression of the inhaled material toward the periphery of the lung [20]. However, in the present study no detectable PF silica levels were found either in non-exposed patients or half of the exposed cases. This could be due to the silica only staying in PF for a limited period of time. Song et al. hypothesized that, in some cases, silica nanoparticles are small enough to pass through the lung tissue into the pleural cavity, yet large enough to block proper lymphatic drainage of pleural membranes and cause PEs [21]. They also warned that a traditional cotton mask usually has apertures of 100–300 μm in diameter, which is much larger than nanoscale, implying that using it to avoid nanomaterial exposure would be ineffective [21].

Our study has limitations. First, the phone survey was sometimes done several years after PE diagnosis and thus patients may have forgotten any previous environmental exposure. Second, the exposure intensity was not investigated. Third, in most cases clinicians were unaware that PEs could be related to occupational exposure and, therefore, pleural or lung biopsies were not properly managed to be analyzed by electron microscopy and EDX. In fact, in only one of our cases were the pleural and lung biopsies suitable for EDX analysis. A case-control, rather than a cohort or cross-sectional design, was performed because it is more efficient for hypothesis generation in a preliminary study with such a small sample size. Moreover, case-control studies permit assessment of the influence of predictors under investigation (silica inhalation in our study) via calculation of odds ratios. However, this specific design is prone to sampling bias and the conclusions should be confirmed with other prospective cohort studies [22]. Finally, the plasma atomic emission spectrometry analysis used to detect silica in the PF is sensitive, but it is not capable of determining their different types. Additionally, this method was tested in a small number of randomly selected patients.

In conclusion, data from the current study suggests that the origin of some undiagnosed PEs could be due to occupational silica exposure. Further pleural tissue analyses with sensitive methods to detect silica, such as EDX, in the context of idiopathic PEs with a history of potential occupational exposure, or cohort studies based on the occupational exposure of the patients, would help to confirm our hypothesis.

## Supplementary Material

Supplementary Material

Supplementary Material
