# Supplementary material for: Some pleural effusions labeled as idiopathic could be produced by the inhalation of silica
Source: Pleura Peritoneum. 2022 Jan 3;7(1):27–33. doi: 10.1515/pp-2021-0135 (PMC9069498; doi:10.1515/pp-2021-0135)
Supplement: Supplementary file 1 — Supplementary Material [file j_pp-2021-0135_suppl_001.docx]

**Annex 1** **Questionnaire about working life**

The purpose of this form is to find out whether some occupations, more than others, are frequently associated with some diseases or if toxic particles could be more often present in some occupational environments.

You have suffered a pleural disease and, if you consent, we invite you to participate by filling a simple questionnaire about your work life that can be completed by telephone.

-Sex: man / woman

-Age when you suffered the pleural disease?

-What jobs have you had and what kind of work have you done?

(For example: construction industry working as a bricklayer)

-How long did you perform each kind of work?

-Specifically, did have you been exposed to any of the following materials (job or hobby)?: sand, crushed stone, paints, ceramics, abrasive powders, cements, industrial or paint aerosols

-Time of exposure for each product (for example, number of months or years).

-Use of protective measures such a breathing masks: time and type of masks.

We considered a worker to have been exposed if he/she belonged to a group of skilled laborers (bricklayer/carpenter/mechanic/miner/painter) or had been exposed to any materials that could contain silica (sand, crushed stone, paints, ceramics, abrasive powders, cements or industrials or paint aerosols).

The interviewer did not know the cause of the pleural effusion. Participants provided verbally informed consent.
