# Supplementary material for: Some pleural effusions labeled as idiopathic could be produced by the inhalation of silica
Source: Pleura Peritoneum. 2022 Jan 3;7(1):27–33. doi: 10.1515/pp-2021-0135 (PMC9069498; doi:10.1515/pp-2021-0135)
Supplement: Supplementary file 2 — Supplementary Material [file j_pp-2021-0135_suppl_002.docx]

**Annex 2 Plasma atomic emission spectrometry procedure**

**Reagents**

Tetramethylammonium hydroxide (TMAH, 25% w/w) aqueous solution was supplied by Alfa Aesar (Massachussetss, EEUU). Two-propanolol HPLC (99.7% grade) and hydrogen peroxide (30% w/w) were obtained from Panreac (Barcelona, Spain). Ultrapure water (resistivity = 18.2 MΩ cm^-1^ at 25°C) was produced in the laboratory by a Milipore Milli-Q-system. Silicon standard solutions were prepared daily from a 1.0 mg ml^-1^ stock solution and afterwards were employed for recovery studies.

**Apparatus**

For the microwave digestion of the samples, a CEM Mars microwave oven providing 1600W of output power (CEM, Cologno al Serio, Italy), equipped with 12 TFM polytetrafluoroetlylene (PTFE) vessels (100 ml, EasyPrep) and temperature and pressure regulation through a sensor vessel was used. Measurements were performed by an inductively coupled plasma- optical emission spectrometry (ICP-OES) IRIS intrepid radial thermo element equipped with a standard ICP torch, crossflow nebulizer and a CID detector.

PTFE vessels, micropipette tips and polypropylene (PP) tubes were cleaned in 10% HNO3 overnight and rinsed thoroughly with ultrapure water.

**Sample collection**

PF samples were collected immediately after thoracenteses into PP tubes containing 5 ml ethylenediaminetetraacetic acid, centrifuged and stored in aliquots at -80°C until assayed. Before use, all samples were thawed and then homogenized by vortex.

**Procedure**

One PF aliquot of each patient were thawed to analyze. PF (1g) was exactly weighed into 100-ml TFM PTFE tubes; and 6ml of TMAH, 2 drops of 2-propanolol, 1ml of H2O2 and 3ml of ultrapure water were subsequently added. Microware vessels were sonicated (15 minutes, 60°C) and irradiated for 15 minutes at 180°C after a temperature ramp of 20 minutes. The clear solutions obtained from digestion were left to cool, diluted to 25ml calibrated PP tubes and analyzed by ICP-OES. The operation conditions and parameters of the ICP-OES were the following: radio-frequency-power 1000W; gas: argon; plasma gas: 15L/min; auxiliary gas: 0.5L/min; nebulizer gas: 0.55L/min; sample aspiration rate: 1mL/min; view: radial; number of replicates: 3; nebulizer: Meinhard (Golden, EEUU); detector: CID.
